# Modified water vapor thermal therapy for large-volume benign prostatic hyperplasia

**DOI:** 10.1186/s12894-026-02050-3

**Published:** 2026-02-07

**Authors:** Qun Lu, Guanchen Zhu, Jun Liu, Haifeng Huang, Fan Zhang, Xuefeng Qiu, Linfeng Xu, Hongqian Guo

**Affiliations:** 1 Department of Urology, Affiliated Drum Tower Hospital, Medical School of Nanjing University, Institute of Urology, Nanjing University, 321 Zhongshan Road, Nanjing, Jiangsu 210008 China; 2 Department of Urology, the Second Affiliated Hospital, Hengyang Medical School, University of South China, Hengyang, Hunan China

**Keywords:** Water vapor thermal therapy, BPH, Large gland, LUTS

## Abstract

**Background:**

This study aims to evaluate the efficacy and safety of modified water vapor thermal therapy for large-volume benign prostatic hyperplasia (BPH).

**Methods:**

This prospective study enrolled 196 consecutive patients with prostate volume ≥ 80 mL who underwent modified water vapor thermal therapy at our institution between October 2023 and September 2024. All procedures were performed using the Rezum system.

**Results:**

The procedures were successfully completed in all 196 patients with a median prostate volume of 96 mL. The IPSS decreased from a preoperative mean of 21.2 ± 3.9 to 11.7 ± 3.4 at 3 months, and further improving to 10.8 ± 3.8 at 1 year. Median prostate volume reduced from 96 mL to 60 mL. The QoL score improved from 4.3 ± 0.9 to 1.7 ± 1.1, and Qmax increased from 8.3 ± 2.3 mL/s to 15.7 ± 3.5 mL/s at 3 months. All observed improvements were statistically significant compared to baseline (*P* < 0.01). The IIEF-5 score increased from 11.0 ± 3.6 to 14.1 ± 5.4 at 1 year, indicating a statistically significant improvement in erectile function (*P* < 0.05). The incidence of retrograde ejaculation was 3.1%. Only one patient (0.5%) required surgical retreatment during the follow-up period.

**Conclusions:**

Modified water vapor thermal therapy demonstrates favorable efficacy and safety in the treatment of large-volume BPH. It is associated with significant symptom relief, functional improvement, and minimal complications.

## Background

 Benign prostatic hyperplasia (BPH) is the most prevalent benign condition contributing to lower urinary tract symptoms (LUTS) in middle-aged and elderly men, with prevalence increasing progressively with age [1–3]. Despite the availability of pharmacological therapy, a subset of patients requires surgical intervention to alleviate bladder outlet obstruction caused by BPH. Transurethral resection of the prostate (TURP) remains the gold standard for patients with a prostate volume less than 80 mL. However, TURP is associated with several potential complications, including transurethral resection syndrome, postoperative retrograde ejaculation, erectile dysfunction, and permanent urinary incontinence [4, 5]. Given these risks, there is growing demand for ultra-minimally invasive therapeutic options that minimize complication while maintaining clinical efficacy. The Rezum system utilizes convective thermal energy delivered via high-temperature water vapor to induce targeted necrosis of hyperplastic prostate tissue while preserving surrounding urethral architecture. This modality has demonstrated symptom relief comparable to traditional transurethral surgery, with a more favorable safety profiles [6, 7].

Current guidelines from the American Urological Association (AUA) recognize water vapor thermal therapy as a viable treatment option for patients with prostate volumes < 80 mL [8]. However, no formal recommendation is provided for individuals with larger prostates. Therefore, this study aimed to evaluate the efficacy and safety of modified water vapor thermal therapy in patients with large-volume BPH.

## Methods

### General information

A total of 196 consecutive BPH patients aged > 50 years, with baseline International Prostate Symptom Score (IPSS) ≥ 13, maximum urinary flow rate (Qmax) ≤ 15 mL/s, and prostate volume ≥ 80 mL, were enrolled between October 2023 and September 2024. All patients completed follow-up assessments over a period of 12 to 23 months.

Procedures were performed using the Rezum system (Boston Scientific Corporation, USA) [9]. The number of injections was determined based on individual prostate volume and the extent of median lobe hyperplasia. Personalized ablation strategies were developed according to prostate size, morphology, and imaging findings, including preoperative magnetic resonance imaging (MRI) and intraoperative transrectal color Doppler ultrasound. Urodynamic testing was performed in all patients to exclude concomitant bladder dysfunction. The study was approved by the local ethics committee, and written informed consent was obtained from all participants.

### Modified water vapor thermal therapy technique

**Fig. 1:**
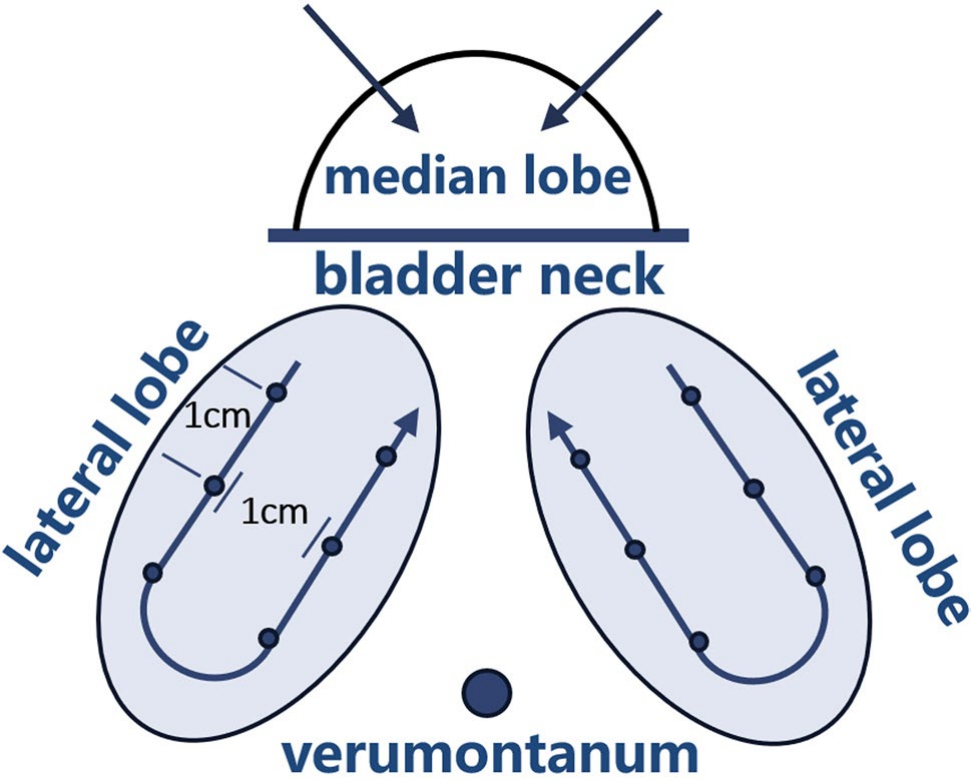
Illustration of the injection method of modified water vapor thermal therapy

Under general or local anesthesia, patients were placed in the lithotomy position. During the procedure, the anteroposterior diameter (from the bladder neck to the verumontanum) and superior-inferior diameter of the prostatic urethra were measured under cystoscopic visualization. Each cystoscopic field of view corresponded to approximately 0.5 cm of tissue length. The total distance was calculated based on the number of fields observed, which informed the number of treatment needles required per row. At least two rows of injections were administered according to the superior-inferior dimension of the gland, with injection sites positioned away from the verumontanum. Special attention was given to the assessment of the median lobe, including its anteroposterior diameter and arc length. U-path injection method was adopted for lateral lobes and V-shaped injection method for middle lobe to ensure complete ablation of the glands (Fig. 1).

**Fig. 2:**
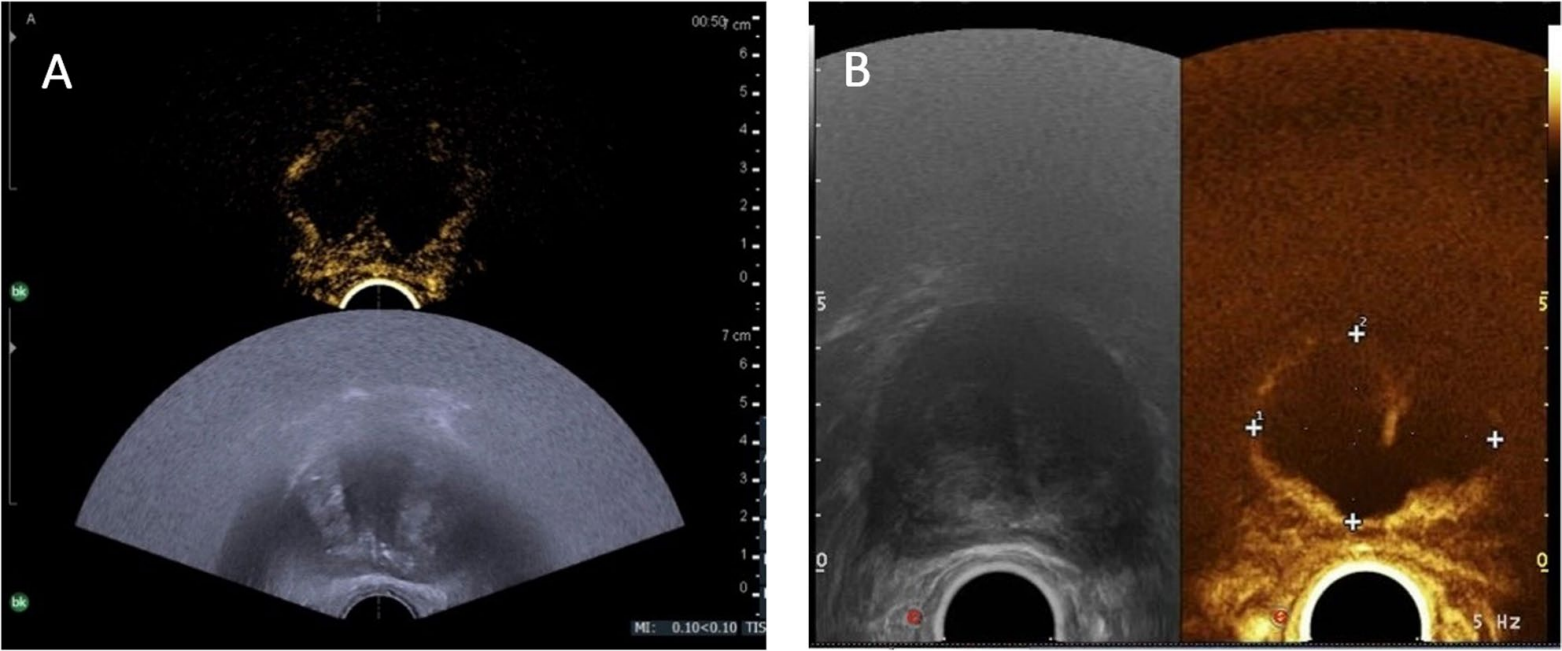
Immediate contrast-enhanced ultrasound of the prostate after modified water vapor thermal therapy (**A**). Contrast-enhanced ultrasound of the prostate at 1 month after modified water vapor thermal therapy (**B**)

Intraoperative ultrasound was used to identify critical anatomical landmarks such as the bladder neck, urethral sphincter, and rectum, ensuring precise targeting and avoidance of adjacent structures. Immediately after ablation, contrast-enhanced ultrasound was performed to evaluate the extent of tissue ablation. A dose of 1.2–2.4 mL of sulfur hexafluoride microbubbles (SonoVue, Bracco, Italy) was injected intravenously via an antecubital vein followed by a 5-mL saline flush. Harmonic microbubble-specific imaging with low acoustic ultrasound pressure was performed by using the Pro Focus 2202 ultrasonography device (BK, Denmark). No focal enhancement within the treated lesion was regarded as technical success, indicating complete ablation of the hyperplastic gland in the transition and central zone (Fig. 2). Additional injections were administered in the event of local perfusion within the gland, and the needle is inserted into the enhanced gland under transrectal ultrasound guidance.

### Assessment

Patients were routinely evaluated at 1, 3, 6, and 12 months post-treatment. Additional assessments beyond 12 months were conducted if persistent or recurrent urinary symptoms were reported. Data collection included demographic characteristics, prostate volume, urinary flow parameters, and validated outcome measures: IPSS, Quality of Life (QoL) score, International Index of Erectile Function-5 (IIEF-5), and need for retreatment. Surgical-related variables recorded included operative time, postoperative hospital stay, duration of indwelling catheterization, and adverse events. All BPH-related medications were discontinued for a washout period of at least one week prior to obtaining baseline outcome measurements and the procedure. After the procedure, medication treatment was initiated in accordance with the severity of LUTS. Complications were classified according to the modified Clavien-Dindo system [10].

### Statistical method

Statistical analysis was performed using SPSS version 21.0 (SPSS Inc., Chicago, IL, USA). Continuous data were expressed as mean ± standard deviation (SD) or median (interquartile range [IQR]), and categorical variables were reported as frequencies and percentages. Between-group comparisons were conducted using independent samples *t* test or Mann-Whitney *U* test, depending on the normality of data distribution. Pearson correlation analysis was conducted to evaluate the association between two variables. Analyses were restricted to patients with both baseline and follow-up assessment values; those missing either measurement were excluded. A two-sided P value < 0.05 was considered statistically significant.

## Results

A total of 196 patients were included in this study, with a median age of 73 years (IQR: 65–79) (Table 1). Preoperative MRI revealed a median prostate volume of 96 mL (IQR: 89.0–105.0). A hyperplastic median lobe was present in 49.5% of patients. The mean preoperative IPSS was 21.2 ± 3.9, and the QoL score was 4.3 ± 0.9. The baseline Qmax was 8.3 ± 2.3 mL/s, and the IIEF-5 score was 11.0 ± 3.6.

**Table 1:** Baseline and perioperative characteristics

Baseline and perioperative characteristics	
Age, years, median (IQR)	73 (65, 79)
Prostate volume, mL, median (IQR)	96.0 (89.0, 105.0)
Median lobe, n (%)	97 (49.5)
Previous BPH surgery, n (%)	
TURP	5 (2.6)
TUSP	1 (0.5)
PAE	3 (1.5)
BPH medication, n (%)	
Alpha-blocker	176 (89.8)
5ARI	143 (73.0)
None	18 (9.2)
Medical history, n (%)	
Hypertension	105 (53.6)
Diabetes	62 (31.6)
Bladder stone	15 (7.7)
History of urinary retention	41 (20.9)
IPSS, mean (SD)	21.2 (3.9)
IPSS QoL, mean (SD)	4.3 (0.9)
Qmax, mL/s, mean (SD)	8.3 (2.3)
IIEF-5, mean (SD)	11.0 (3.6)
Anaesthesia, n (%)	
General anesthesia	179 (91.3)
Local anesthesia	17 (8.7)
Number of injections, median (IQR)	13 (10, 15)
Left lobe	6 (5, 7)
Right lobe	6 (5, 7)
Median lobe	1 (0, 2)
Operative time, min, median (IQR)	9 (6, 10)
Catheter duration, days, median (IQR)	14 (10, 14)
Postoperative medications, n (%)	
Antibiotics	193 (98.5)
Pain medications	71 (36.2)
Alpha-blocker	53 (27.0)
β3-adrenergic agonist	68 (34.7)
Retrograde ejaculation, n (%)	6 (3.1)
Retreatment need, n (%)	1 (0.5)

*TURP* Transurethral resection of the prostate, *TUSP* Transurethral split of the prostate, *PAE* Prostatic artery embolization, *5ARI* 5-alpha reductase inhibitor, *IPSS* International Prostate Symptom Score, *QoL* Quality of life, *Qmax* maximum urinary flow rate, *IIEF-5* International Index of Erectile Function-5, *SD* Standard deviation, *IQR* Interquartile range

All 196 procedures were successfully completed. The median number of injections was 6 (IQR: 5–7) in the left lobe, 6 (IQR: 5–7) in the right lobe, and 1 (IQR: 0–2) in the median lobe. No significant intraoperative bleeding was observed at any puncture site. An F18 silicone catheter was placed postoperatively. The median operative time was 9 min (IQR: 6–10), and the median duration of catheterization was 14 days (IQR: 10–14).

**Fig. 3:**
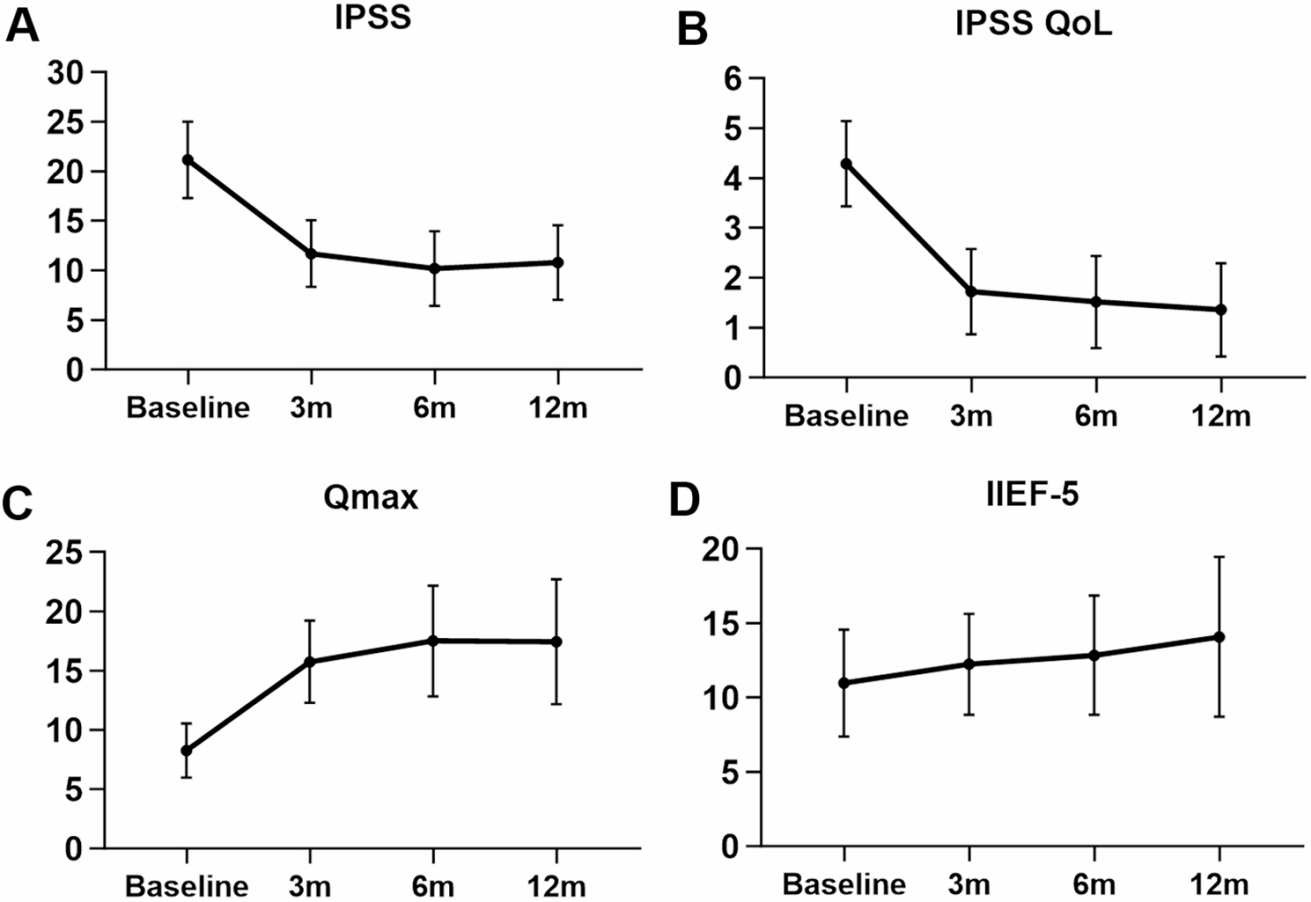
Graphical representation of outcomes for modified water vapor thermal therapy. **A**, IPSS. **B**, IPSS QoL. **C**, Qmax. **D**, IIEF-5

**Fig. 4:**
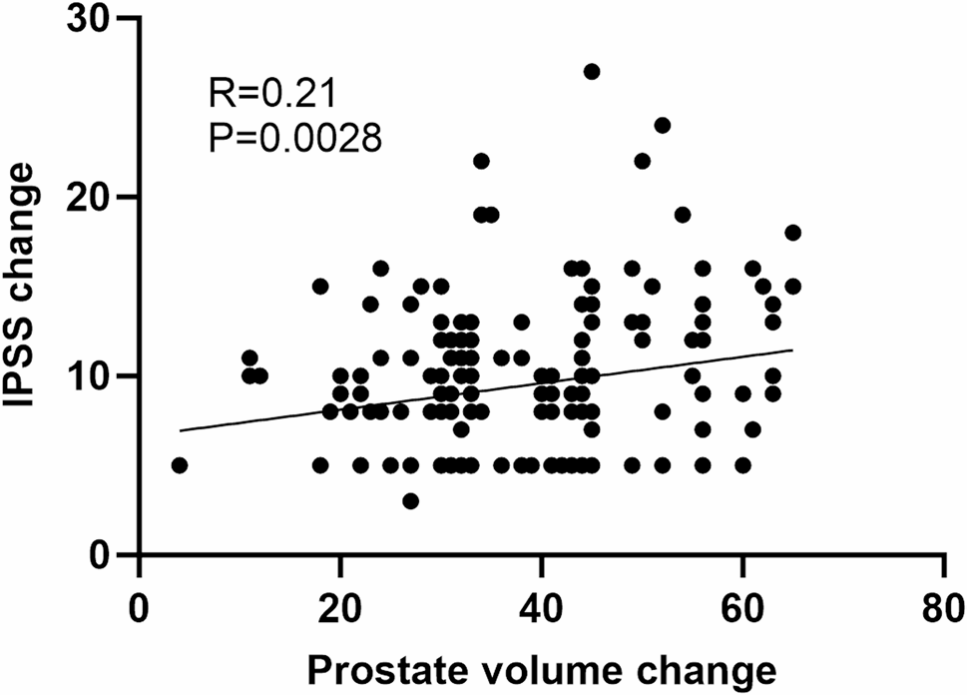
Pearson correlation analysis between changes in prostate volume and changes in IPSS at 3 months

Patients were followed up for 12 to 23 months postoperatively. The IPSS decreased from a preoperative mean of 21.2 ± 3.9 to 11.7 ± 3.4 at 3 months and further improved to 10.8 ± 3.8 at 1 year. The QoL score improved from 4.3 ± 0.9 to 1.7 ± 1.1, and Qmax increased from 8.3 ± 2.3 mL/s to 15.7 ± 3.5 mL/s at 3 months. All these improvements were statistically significant compared to baseline values (*P* < 0.01). The IIEF-5 score increased from 11.0 ± 3.6 to 14.1 ± 5.4 at 1 year, indicating a statistically significant improvement in erectile function (*P* < 0.05) (Fig. 3). The incidence of retrograde ejaculation was 3.1%. Median prostate volume reduced from 96 mL to 60 mL. Pearson correlation analysis revealed a significant association between changes in prostate volume and changes in IPSS at 3 months (Fig. 4). Only one patient (0.5%) required surgical retreatment during the follow-up period.

## Discussion

Surgical intervention is indicated in patients present with recurrent urinary retention, hematuria, bladder stones, or secondary upper urinary tract hydronephrosis. Minimally invasive transurethral surgery currently serves as the primary approach [11–13]. However, conventional minimally invasive transurethral techniques are associated with risks such as urinary incontinence, retrograde ejaculation, and erectile dysfunction, particularly among elderly patients with reduced tolerance. A substantial proportion of elderly men with BPH remain in need of safe and effective treatment options [14]. Water vapor thermal therapy offers an ultra-minimally invasive alternative for the management of BPH, suitable for day-case or outpatient settings. It is characterized by short operative times and rapid recovery, effectively alleviating clinical symptoms and improving quality of life without compromising sexual function [15, 16].

In contrast to earlier thermal ablation methods such as microwave thermotherapy, which rely on conductive heat transfer, water vapor thermal therapy utilizes convective heat transfer. This mechanism avoids temperature gradients and minimizes thermal damage to tissues surrounding the targeted treatment zone [17]. The findings of this study indicate that following treatment with the Rezum system, the IPSS decreased from a baseline of 21.2 ± 3.9 points to 11.7 ± 3.4 points at 1 year, reflecting a reduction of 10.4 ± 4.7 points. According to the Rezum Pivotal trial, the experimental group exhibited a decrease in IPSS of 11.2 points at 3 months postoperatively [6]. Comparable urinary improvement effects were observed in large-volume prostates in the present study. Water vapor thermal therapy leads to sustained improvement in LUTS, with reported IPSS reductions ranging from 47% to 60% [18]. It should be noted that these prior studies primarily included patients with prostate volumes between 30 and 80 ml.

Currently, evidence regarding water vapor thermal therapy for large-volume prostate glands remains limited, highlighting the need for further investigation [19, 20]. Achieving adequate ablation in large-volume prostates represents a significant clinical challenge [21]. In this study, water vapor thermal therapy was modified to better address large-volume prostates. Multiple rows of injections were administered according to the superior-inferior dimension of the gland. Intraoperative ultrasound was utilized to identify critical anatomical structures including the bladder neck, urethral sphincter, and rectum. Contrast-enhanced ultrasound enabled evaluation of the ablation zone and helped determine whether additional injections were required. No serious adverse events, such as rectal or bladder perforation, occurred during the procedure. The most frequently reported adverse events were urethral discomfort, distending pain, and hematuria, supporting the favorable safety profile of the water vapor thermal therapy.

This study has several limitations, including its single-arm design, and lack of a control group. Furthermore, longer-term follow-up studies are needed to validate the durability of clinical outcomes following water vapor thermal therapy in patients with large prostates.

## Conclusions

Modified water vapor thermal therapy provides significant alleviation of LUTS, improves quality of life, and preserves sexual function in patients with large-volume BPH, while demonstrating a favorable safety profile.

## Data Availability

The datasets used and analyzed during the current study are available from the corresponding author on reasonable request.

## Abbreviations

BPH: Benign prostatic hyperplasia
LUTS: Lower urinary tract symptoms
TURP: Transurethral resection of the prostate
IPSS: International Prostate Symptom Score
QoL: Quality of Life
IIEF-5: International Index of Erectile Function-5

## References

[1] Chughtai B, Forde JC, Thomas DD, et al. Benign prostatic hyperplasia. Nat Rev Dis Primers. 2016;2:16031. 27147135 10.1038/nrdp.2016.31

[2] Baug S, Beisland C, Moen CA, et al. Transurethral resection of the prostate in the extreme elderly (>/= 85 years): treatment success, morbidity and survival. World J Urol. 2025;43(1):572. 40991051 10.1007/s00345-025-05948-z PMC12460449

[3] Pirola GM, Castellani D, Naselli A, et al. Endoscopic enucleation of the prostate in men aged 80 years and older. Outcomes from a global, large, and multicenter series using different energy sources and techniques. World J Urol. 2025;43(1):344. 40448729 10.1007/s00345-025-05699-x

[4] Lee SWH, Chan EMC, Lai YK. The global burden of lower urinary tract symptoms suggestive of benign prostatic hyperplasia: A systematic review and meta-analysis. Sci Rep. 2017;7(1):7984. 28801563 10.1038/s41598-017-06628-8 PMC5554261

[5] Jin Q, Zhang J, Wang G, et al. The endoscopic surgical monitoring system-guided impact factors exploring of blood loss and fluid absorption during plasmakinetic TURP: a prospective study in high-risk BPH patients. BMC Urol. 2025;25(1):287. 41254640 10.1186/s12894-025-01970-w PMC12625336

[6] McVary KT, Gittelman MC, Goldberg KA, et al. Final 5-Year outcomes of the multicenter randomized Sham-Controlled trial of a water vapor thermal therapy for treatment of moderate to severe lower urinary tract symptoms secondary to benign prostatic hyperplasia. J Urol. 2021;206(3):715–24. 33872051 10.1097/JU.0000000000001778

[7] Cornu JN, Zantek P, Burtt G, et al. Minimally invasive treatments for benign prostatic obstruction: A systematic review and network Meta-analysis. Eur Urol. 2023;83(6):534–47. 36964042 10.1016/j.eururo.2023.02.028

[8] Management of Lower Urinary Tract Symptoms Attributed to Benign Prostatic Hyperplasia. AUA guideline PART II-Surgical evaluation and treatment. Erratum J Urol. 2022;207(3):743. 10.1097/JU.0000000000002388 35135337

[9] McVary KT, Gange SN, Gittelman MC, et al. Minimally invasive prostate convective water vapor energy ablation: A Multicenter, Randomized, controlled study for the treatment of lower urinary tract symptoms secondary to benign prostatic hyperplasia. J Urol. 2016;195(5):1529–38. 26614889 10.1016/j.juro.2015.10.181

[10] Dindo D, Demartines N, Clavien PA. Classification of surgical complications: a new proposal with evaluation in a cohort of 6336 patients and results of a survey. Ann Surg. 2004;240(2):205–13. 15273542 10.1097/01.sla.0000133083.54934.ae PMC1360123

[11] Wei JT, Dauw CA, Brodsky CN. Lower urinary tract symptoms in men: A review. JAMA. 2025;334(9):809–21. 40658396 10.1001/jama.2025.7045

[12] Balsamo R, Tammaro S, Fusco F, et al. Patients with frailty, benign prostatic hyperplasia and indwelling bladder catheter: what are the 1-year outcomes after Rezum therapy?. Curr Urol. 2025;19(6):396–400. 41058769 10.1097/CU9.0000000000000295 PMC12499839

[13] Giulioni C, Talle M, Papaveri A, et al. The implementation of trifecta score to assess the quality of holmium laser enucleation of the prostate in elderly patients: an analysis of perioperative and functional outcomes and the impact of age. J Clin Med. 2025;14(10):3410. 10.3390/jcm14103410 PMC12112108 40429404

[14] Elterman D, Gao B, Lu S, et al. New technologies for treatment of benign prostatic hyperplasia. Urol Clin North Am. 2022;49(1):11–22. 34776045 10.1016/j.ucl.2021.07.007

[15] Westwood J, Geraghty R, Jones P, et al. Rezum: a new transurethral water vapour therapy for benign prostatic hyperplasia. Ther Adv Urol. 2018;10(11):327–33. 30344644 10.1177/1756287218793084 PMC6180381

[16] Balsamo R, Tammaro S, Trivellato M, et al. Water vapor thermal therapy (Rezum System) in patients with large prostates: results from a prospective comparative study. Minerva Urol Nephrol. 2024;76(6):759–67. 39494943 10.23736/S2724-6051.24.05883-X

[17] Yang J, Wu W, Amier Y, et al. Efficacy and safety of water vapor thermal therapy in the treatment of benign prostate hyperplasia: a systematic review and single-arm Meta-analysis. BMC Urol. 2023;23(1):72. 37118692 10.1186/s12894-023-01237-2 PMC10147364

[18] Doppalapudi SK, Gupta N. What is new with Rezum water vapor thermal therapy for LUTS/BPH?. Curr Urol Rep. 2021;22(1):4. 33403529 10.1007/s11934-020-01018-6

[19] McVary KT, Miller LE, Bhattacharyya S, et al. Water vapor thermal therapy in men with prostate volume >/=80 cm(3): A systematic review and Meta-Analysis. Urology. 2024;184:244–50. 38006957 10.1016/j.urology.2023.10.036

[20] Elterman D, Bhojani N, Vannabouathong C, et al. Rezum therapy for >/=80-mL benign prostatic enlargement: a large, multicentre cohort study. BJU Int. 2022;130(4):522–7. 35466513 10.1111/bju.15753

[21] Dixon CM, Rijo Cedano E, Mynderse LA, et al. Transurethral convective water vapor as a treatment for lower urinary tract symptomatology due to benign prostatic hyperplasia using the Rezum((R)) system: evaluation of acute ablative capabilities in the human prostate. Res Rep Urol. 2015;7:13–8. 25674555 10.2147/RRU.S74040 PMC4321608

